# Plant-Based Antioxidants for Prevention and Treatment of Neurodegenerative Diseases: Phytotherapeutic Potential of *Laurus nobilis*, *Aronia melanocarpa*, and Celastrol

**DOI:** 10.3390/antiox12030746

**Published:** 2023-03-18

**Authors:** Kristina Pilipović, Renata Jurišić Grubešić, Petra Dolenec, Natalia Kučić, Lea Juretić, Jasenka Mršić-Pelčić

**Affiliations:** 1 Department of Basic and Clinical Pharmacology and Toxicology, Faculty of Medicine, University of Rijeka, HR-51000 Rijeka, Croatia; 2 Department of Physiology and Immunology, Faculty of Medicine, University of Rijeka, HR-51000 Rijeka, Croatia

**Keywords:** neurodegenerative diseases, oxidative stress, neuroinflammation, *Laurus nobilis*, *Aronia melanocarpa*, celastrol

## Abstract

With the progress of medicine, especially in the last century, life expectancy increased considerably. As a result, age-related diseases also increased, especially malignancies and degenerative diseases of the central nervous system. The incidence and prevalence of neurodegenerative diseases steadily increased over the years, but despite efforts to uncover the pathophysiological processes behind these conditions, they remain elusive. Among the many theories, oxidative stress was proposed to be involved in neurodegenerative processes and to play an important role in the morbidity and progression of various neurodegenerative disorders. Accordingly, a number of studies discovered the potential of natural plant constituents to have significant antioxidant activity. This review focused on several plant-based antioxidants that showed promising results in the prevention and treatment of neurodegenerative diseases. *Laurus nobilis*, *Aronia melanocarpa*, and celastrol, a chemical compound isolated from the root extracts of *Tripterygium wilfordii* and *T. regelii*, are all known to be rich in antioxidant polyphenols.

## 1. Introduction

Neurodegenerative diseases (NDDs) are disorders characterized by the progressive loss of neuronal cell structure and function. The incidence of NDDs increases over the years due to increased life expectancy [1]. NDDs are generally considered age-related diseases, but can also affect the younger population [2]. Despite decades of preclinical and clinical research aimed at uncovering the processes underlying the development of NDDs, the pathogenesis remains largely unknown. Evidence suggests that both genetic predisposition and environmental factors play an important role [3].

Depending on which group of neurons degenerates, specific diseases and symptoms occur. The most common NDDs are Alzheimer’s disease (AD), Parkinson’s disease (PD), Huntington’s disease (HD), amyotrophic lateral sclerosis (ALS), multiple sclerosis, and other less common diseases [2]. AD is characterized by memory loss, cognitive impairment, confusion, aggression, and mood swings, leading to the progressive deterioration of behavior and functionality. Key pathological features of AD include extracellular deposits of insoluble beta-amyloid (Aβ) plaques and intracellular accumulations of neurofibrillary tangles composed of hyperphosphorylated tau protein [4,5,6]. Regional neuron loss is found in the medial temporal lobe, hippocampus, and cerebral cortex, with cholinergic pathways particularly affected [7]. PD is characterized by selective loss of dopaminergic neurons in the supstantia nigra pars compacta, resulting in decreased dopamine levels in the nigrostriatal pathway. Consequently, motor disturbances such as resting tremor, muscle rigidity, bradykinesia, and postural instability are typical clinical manifestations of the disease. The pathological feature of PD is the presence of Lewy bodies, which are aggregates of various proteins such as α-synuclein, ubiquitin, parkin, and neurofilaments. HD is a genetic disease that leads to the progressive degeneration of neurons in the striatum, cerebral cortex, and thalamus, resulting in a steady progression of motor dysfunction. ALS is characterized by the progressive degeneration of motor neurons in the spinal cord, brainstem, and cortex, resulting in spasticity, muscle weakness, progressive paralysis, dysphagia, and respiratory insufficiency [7]. The main pathological feature of ALS is the mislocalization of proteins and the formation of ubiquitinated cytoplasmic aggregates in degenerating neurons and their surrounding oligodendrocytes. Although certain NDDs have their own specific features, they are all irreversible, progressive, and without effective therapeutic options.

Currently available treatments can help alleviate symptoms and slow disease progression, but there is no therapeutic option to prevent or cure NDDs.

The molecular pathogenesis of NDDs is still controversial, but evidence suggests that it involves a complex interplay between genetic disorders, environmental factors, misfolding and aggregation of various proteins, disturbances in metal ion homeostasis, mitochondrial dysfunction, excitotoxicity, neuroinflammation, oxidative stress (OS), and the activation of programmed cell death [2,4,7,8,9]. There is increasing evidence that OS plays a central role in the development of NDDs [2,10,11,12]. Moreover, it may be a very important factor in secondary neurodegenerative changes observed after CNS injuries such as ischemia or traumatic brain injury [13].

Oxidative and nitrosative stress are the result of an imbalance between the generation of two types of reactive molecules, reactive oxygen species (ROS) and reactive nitrogen species (RNS), on the one hand, and the balancing antioxidant mechanisms on the other [14]. In general, ROS/RNS have some useful functions, but their accumulation may trigger the activation of pathophysiological pathways leading to the development of NDDs via different mechanisms [7,11]. Elevated levels of reactive species can damage cellular components such as lipids, proteins, and DNA, leading to neuronal dysfunction and death [11]. Therefore, the search for therapeutic agents that could influence this pathological cascade by modulating the production of reactive species and/or antioxidant mechanisms seems to be a promising strategy.

Throughout history, people around the world used medicinal plants and their preparations to improve health. Today, many plant species already have an established place in scientific medicine and are used in the treatment of a wide range of health conditions, including NDDs. However, the golden field of medicinal plants still hides many secrets and is a great challenge for modern scientific research in discovering new potential phytotherapeutics [15].

Both synthetic and natural plant products with antioxidant properties are being explored as potential treatments for NDDs [16,17,18]. Natural plant products recently gained attention, as they were shown to have neuroprotective effects and reduce OS and inflammation [1,19]. Natural antioxidants are found in fruits, vegetables, nuts, and herbs, and include compounds such as polyphenols, carotenoids, and vitamins C and E. Some examples of plant-based antioxidants that were extensively studied are polyphenols from green tea, resveratrol from grapes, and curcumin from turmeric, etc. [7,16,20,21,22]. Although promising, further research is needed to fully understand their role and possible therapeutic potential in NDDs. In addition, the best dosages and delivery methods need to be determined to ensure their bioavailability.

The aim of this review is to present and discuss several plant-based products that have antioxidant and anti-inflammatory properties, making them promising therapeutic agents for the treatment of NDDs. We focus on the neuroprotective potential of leaf extracts of *Laurus nobilis*, *Aronia melanocarpa* (black chokeberry extracts), and celastrol, a chemical compound isolated from the root extracts of *Tripterygium wilfordii* and *T. regelii*, all known to be enriched with antioxidant polyphenols.

## 2. The Role of Oxidative Stress in the Pathophysiology of Neurodegenerative Diseases

Oxygen is essential to all aerobic organisms because it is the final electron acceptor in the electron transport chain during oxidative phosphorylation in mitochondria, the major energy-producing machinery. The oxidation of glucose is the main energy source of the brain because of its high ATP generation rate, which is required to maintain the high energy demand of neurons [6]. As a result, ROS and RNS are produced. Both groups of reactive species include free radicals, which are extremely reactive chemical species that possess one or more unpaired electrons [14]. Hydroxyl (OH^•^), superoxide (O_2_^•−^), and nitric oxide (NO^•−^) are free radicals, while hydrogen peroxide (H_2_O_2_), nitric oxide (NO), peroxynitrite (ONOO^−^), and hypochlorous acid (HOCl) are not free radicals, but they can generate them through various chemical reactions [4,9]. Although they are mainly produced in mitochondria, there are other sources such as cytochrome P450 enzymes, peroxisomes (by-product of the degradation of fatty acids and other lipid molecules), and phagocytosis of bacteria or viruses [9].

Cells have various safety mechanisms to keep the concentration of reactive species below the level at which they become toxic, as they can cause significant damage to the cell if this threshold is exceeded. These antioxidant systems are responsible for removing and scavenging free radicals and their precursors, inhibiting their formation, and binding metal ions needed to catalyze free radical formation [9]. They can be enzymatic or non-enzymatic. The most important antioxidant enzymes are superoxide dismutase (SOD), glutathione peroxidase (GPx), catalase (CAT), glutathione reductase, and peroxiredoxins [14]. Non-enzymatic antioxidants are mainly exogenous substances derived from food, such as vitamins C and E, carotenoids, polyphenols (e.g., flavonoids, anthocyanins, phenolic acids, quinones, coumarins, tannins), etc. [19,23]. Endogenous non-enzymatic antioxidants include glutathione and coenzyme Q10 [19,24].

ROS/RNS play an important role in physiological processes such as signal transduction, gene transcription, immune response, response to stressors, synaptic plasticity, learning, and memory [6,9,12]. However, when free radical production and detoxification are out of balance, OS results. This may be due to either the excessive production of reactive species or the dysfunctionality of antioxidant systems. Oxygen overload causes oxidative damage to biomolecules (lipids, proteins, DNA) and alters their structure and function, thus initiating a pathophysiological cascade that leads to cell damage and eventual cell death. Persistent OS can lead to the development of various chronic diseases, including aging and degenerative diseases [9]. Indeed, high levels of OS are often observed in many patients diagnosed with NDDs [10,12,19], but it is currently unknown whether OS is a driving force or a consequence that further exacerbates the disease [25].

The brain is particularly susceptible to OS damage because of high oxygen consumption, inadequate antioxidant defense systems, abundant redox-active metals (such as iron and copper), and high levels of polyunsaturated fatty acids (PUFAs), which are susceptible to peroxidation and oxidative changes [6,10]. In addition, glial cells and neurons are postmitotic, highly differentiated cells and are, therefore, particularly sensitive to ROS [9]. Lipids are highly susceptible to free radical attacks and undergo lipid peroxidation, proteins undergo carbonylation and nitration, and nucleic acids are oxidized [6,19]. These processes have complex effects on mitochondria, metal metabolism, and glial cells, causing neuroinflammation and triggering various forms of programmed cell death such as apoptosis, parthanatos, and ferroptosis [19,25].

**Figure 1:**
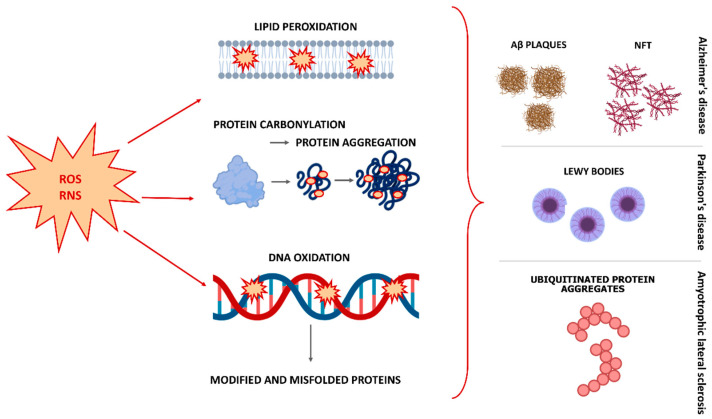
Effects of oxidative stress on lipids, proteins, and DNA, and generation of key pathological features in Alzheimer’s disease, Parkinson’s disease, and amyotrophic lateral sclerosis. Abbreviations: ROS, reactive oxygen species; RNS, reactive nitrogen species; Aβ, beta-amyloid; NFT, neurofibrillary tangles.

Lipid peroxidation is the result of cell membrane damage due to the attack of reactive species on lipids containing carbon-carbon double bonds, especially PUFAs, which are abundant in the brain [6,26]. As a result, a heterogeneous group of relatively stable end products is formed that can bind proteins and DNA, leading to a change in their conformation and function [27,28]. The membrane becomes permeable to substances that are normally unable to cross the barrier, and as a result, these substances cause damage to membrane proteins, enzymes, receptors, and other cell components [19]. Lipoxygenase as well as cyclooxygenase (COX) cascades, which can further impair PUFA metabolism, are upregulated in chronic and age-related brain pathologies [10]. COX-1, COX-2 are responsible for the formation of many eicosanoids, and arachidonic acid and other PUFAs play a critical role in the formation of bioactive lipids, that significantly affect the course of neurodegeneration [10]. The nucleic acids are highly susceptible to oxidative damage. Guanine in particular is more susceptible to attack by ROS, leading to the formation of 8-hydroxyguanine and 8-hydroxy-2-deoxyguanosine. The increased levels of these modified bases were observed in PD brains, for example. In addition, protein carbonylation and nitration were observed in neurodegenerative brains [19]. Oxidative damage results in a variety of oxidative modifications to proteins, as multiple amino acid residues are amenable to oxidation. These modifications can alter cellular function by regulating the stability, activity, subcellular localization, or protein–protein interaction of oxidized proteins [25]. The most important result of protein oxidation is the formation of large protein aggregates, the accumulation of which in cells leads to toxicity. Insoluble aggregates can result from covalent cross-links between peptide chains, as in the case of mutant SOD1 in ALS, α-synuclein in PD, and neurofibrillary tangles and Aβ in AD [10]. The relationship between OS and protein aggregation appears to be bidirectional, as aggregation-prone conformers enhance ROS production, while OS, in turn, promotes protein aggregation [25] (Figure 1).

**Figure 2:**
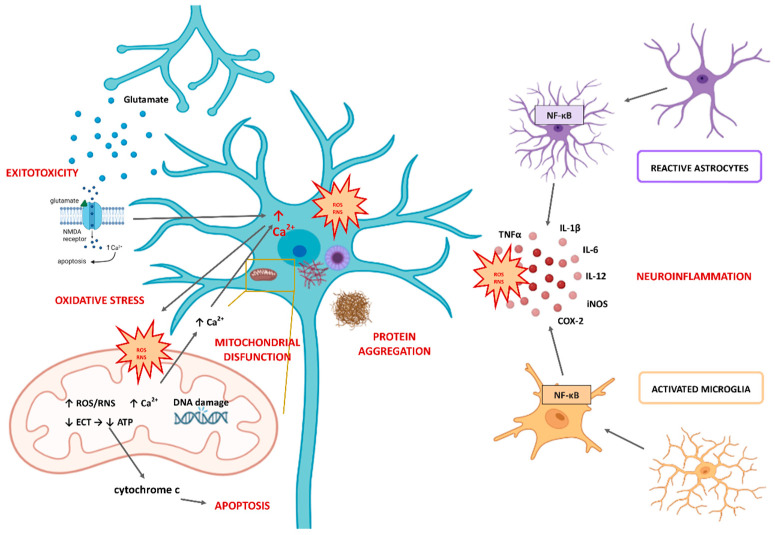
Molecular and cellular mechanisms underlying oxidative stress-stimulated neurodegeneration. Abbreviations: ROS, reactive oxygen species; RNS, reactive nitrogen species; NMDA, N-methyl-D-aspartate receptor; ETC, electron transport chain; NF-κB, nuclear factor-κB; IL-, interleukin-; TNFα, tumor necrosis factor alpha; iNOS, inducible form of nitric oxide; COX-2, cyclooxygenase-2.

Several cellular events such as mitochondrial dysfunction, Ca^2+^ overload, excitotoxicity, and induction in intracellular signaling mechanisms were shown to be major contributors to the molecular and cellular processes underlying OS-stimulated cell death (Figure 2). Some of the most important functions of mitochondria include the production of ATP, regulation of Ca^2+^ homeostasis, control of cell division, and control of cell death by apoptosis [29,30]. Mitochondria are the first to be affected by OS as they are the main source of production of ROS. OS in mitochondria can lead to oxidative damage to mitochondrial membranes, mitochondrial DNA, and mitochondrial proteins, ultimately resulting in mitochondrial damage [10]. Oxidative damage to mitochondrial DNA disrupts the synthesis of respiratory complexes and proteins involved in electron transport, leading to impaired mitochondrial function [24]. This may contribute to a further increase in the production of ROS and the development of OS. Mitochondrial damage may also lead to uncontrolled Ca^2+^ overload because mitochondria play a role in Ca^2+^ homeostasis by secreting excess cytosolic Ca^2+^ in their matrix. In addition, oxidative damage to mitochondria plays a critical role in the release of cytochrome c into the cytosol, where it can activate caspase-dependent apoptosis [24]. Mitochondrial dysfunction is one of the main features of the aging process, especially in organs that require a high energy source, such as the brain [6,18]. Moreover, many of the genes associated with NDDs development are linked to mitochondria [2], and all aggregated misfolded proteins (Aβ, tau, and α-synuclein) are known to inhibit mitochondrial function and induce OS [6,31]. In addition, mitophagy can cause the removal of damaged mitochondria, and recent studies showed that it is involved in the pathological mechanisms of many diseases, especially NDDs and cerebral ischemic diseases [32].

The maintenance of Ca^2+^ homeostasis is a crucial component for preventing neuronal cell death triggered by excitotoxicity. In the brain and neuronal tissue, excitatory amino acids and neurotransmitters are factories for free radicals and ROS [9]. Excessive excitatory stimuli, i.e., excitotoxicity, cause massive calcium influx, membrane depolarization, mitochondrial dysfunction, and subsequent death in neurons. This occurs through the production of reactive species and activation of calcium-dependent pro-death factors, such as calpains.

There is much evidence to suggest that neuroinflammation may be a major factor in the progression of NDDs in addition to OS [5,33] (Figure 2). Inflammatory responses caused by various disorders in the central nervous system (CNS) environment are mediated by glial cells, with microglia playing the central role in neuroinflammation. Microglia promote healthy overall brain physiology by participating in many important processes, such as neuroplasticity and brain remodeling, the release of specific neurotrophic factors, clearance of damaged neurons, response to damage and pathogen-induced stimuli, etc. When glia are activated by certain stimuli, they respond in a pro-inflammatory manner. This response is beneficial for a certain period, but if it is prolonged or becomes abnormal, it becomes maladaptive, and as a result, functional deficits may occur. Mitochondrial dysfunction and OS are thought to cause microglia and astrocytes to become pro-inflammatory and release various cytokines, cytotoxic mediators, and reactive species that increase inflammatory responses in the brain and lead to neuroinflammation. Interleukin (IL)-12, IL-1β, IL-6, nitric oxide (NO), tumor necrosis factor alpha (TNFα) are just some of them. This constant activation of glial cells creates a vicious cycle that promotes chronic neurodegenerative processes [2]. Moreover, nuclear factor kappa B (NF-κB) and mitogen-activated protein kinase (MAPK) are two crucial signaling pathways that regulate the expression of pro-inflammatory cytokines during the inflammatory process in glial cells [33]. As a result, the inducible form of nitric oxide (iNOS), COX-2, and subunits of nicotinamide adenine dinucleotide phosphate (H) (NADPH) oxidase (NOX) are produced, leading to further production of ROS. Altogether, this interplay of OS and neuroinflammation forms a loop of chronic neuronal damage, possibly leading to the development of NDDs. The involvement of the immune system in NDDs is supported by the findings that proinflammatory microglia are closely associated with protein aggregate pathologies characteristic of most dementias [34]. Moreover, microglia spatially correlate with microtubule-associated protein tau pathology and are able to recognize and eliminate tau. They can also phagocytose α-synuclein, the constituent protein of Lewy bodies, becoming proinflammatory by expressing cytokines, ROS, and COX-2 [34].

It is also important to mention nuclear factor erythroid 2-related factor 2 (Nrf2), as its role was reported to be altered in many NDDs [19]. Nrf2 can bind with high affinity to the antioxidant-responsive element (ARE) and contribute to the upregulation of genes involved in modulating cellular redox status and protecting cells from OS [10]. Numerous data support the protective role of the Nrf2 ARE pathway in NDDs by reducing OS and neuroinflammation [19].

In response to stressors such as OS, inflammation, heat stress, and hypoxia, heat shock protein (HSP) expression is upregulated in cells to promote their survival [35]. HSPs are a family of different chaperones named after their molecular mass. They play a role in the proper folding of newly synthesized proteins and proteins subjected to stress-induced denaturation, and some of them also directly interfere with apoptosis [35,36]. These homeostatic functions are particularly important in proteinopathic NDDs, in which certain proteins are misfolded and aggregated. The protective nature of many HSPs was demonstrated in many experimental models of neurodegeneration [36].

The presence of various groups of secondary metabolites, especially phytochemical components such as phenolic compounds and essential oils, were related to health promoting features of medical plants. The most important and widely investigated feature of phenolics is their protective role in OS-induced damage. This protection can cause the delay and/or inhibition of various diseases and degenerative conditions, such as CNS disorders, cardiovascular diseases, atherosclerosis, cancer, diabetes, respiratory, and autoimmune diseases [15].

## 3. *Laurus nobilis* (Lauraceae)

Various plant extracts, especially essential oils (EOs), contain a great number of phytochemicals with diverse physiological effects, including antioxidant, antibacterial, antifungal, antiviral, anticancer, anti-inflammatory, and neurological activities. The use of EOs was known for a long time in traditional medicine and aromatherapy for the treatment of various OS-related disorders. Recently, there was an increased interest in their neuroprotective and anti-aging potentials, as well as for reducing anxiety and stress [37].

**Figure 3:**
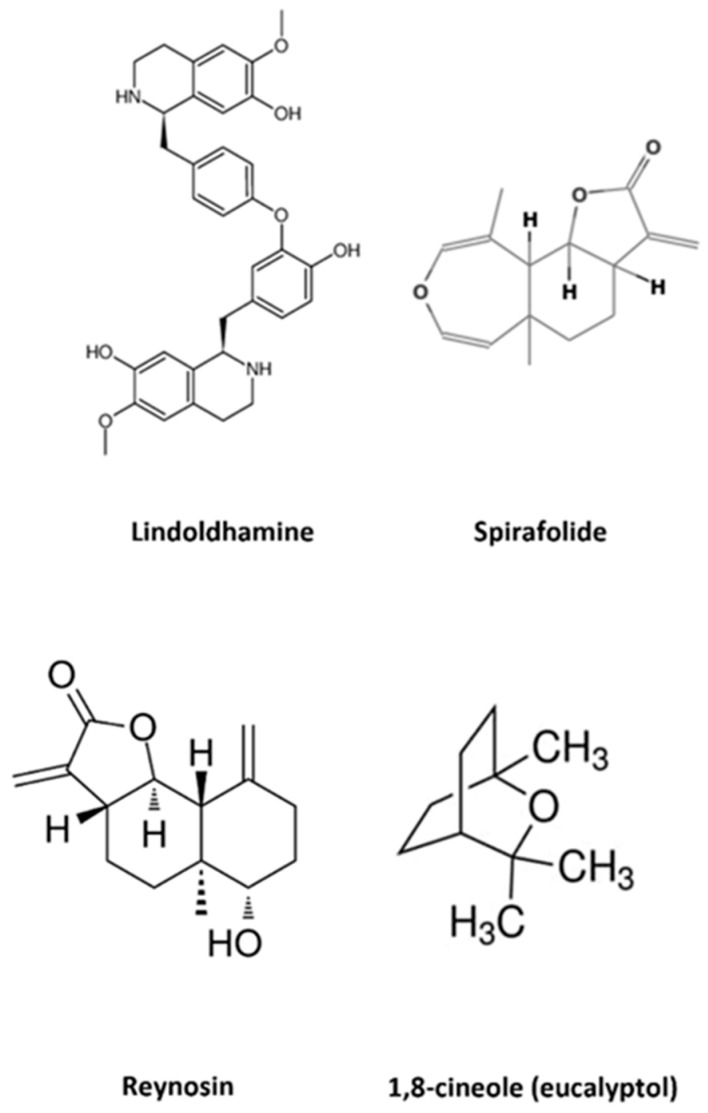
Chemical structures of specific bioactive components of *Laurus nobilis*.

In the countries of the Mediterranean coast, wild and cultivated medicinal plants are widely used in traditional medicine and nutrition. One of these plant species is *Laurus nobilis* L. (Lauraceae), also known as sweet bay, bay laurel, laurel, true bay, or simply bay. It is a perennial evergreen shrub or medium-high tree (10–15 m) cultivated in many warm regions of the world, with natural populations in southern Europe, especially in the Mediterranean region [38].The pharmaceutical properties of the leaves and fruits of *L. nobilis* were known since Dioscorides’s time. Bay laurel leaves were used in folk medicine to treat epilepsy, neuralgia, and parkinsonism [39]. The chemical composition of bay leaves was extensively studied: essential oil constituents, sesquiterpenes, alkaloids, flavonoids, phenolic acids, and lignans were identified [40] (Figure 3). The essential oil extracted from *L. nobilis* leaves (LNEO) exhibited beneficial functions, such as antibacterial, antifungal, and antioxidant activities [41]. Studies on the chemical composition of LNEO generally showed that the main constituents are as follows: 1,8-cineole (eucalyptol), α-terpineol, α-terpinyl acetate, linalool, β-thujene, sabinene, methyl eugenol, α-pinene, and eugenol, with varying concentrations from different locations. LNEO was used for relieving hemorrhoidal and rheumatic pain; it was also found to have diuretic, antifungal, and antibacterial effects. Recent pharmacological studies also demonstrated an anti-acetylcholinesterase property of LNEO [39]. Laurel bay extracts were found to have antioxidant, antibacterial, neuroprotective, and anticholinergic properties [42]. Table 1 presents phytochemical preparations and compounds with neuroprotective potential obtained from bay laurel leaves.

### 3.1. Therapeutic Potential of Laurus nobilis in Neurodegenerative Diseases

#### 3.1.1. *Laurus nobilis* and Alzheimer’s Disease (Anti-Acetylcholinesterase Activity)

Acetylcholinesterase (AChE) is an enzyme significantly involved in NDDs, especially AD. AChE is an important pathogenic factor in AD, as its main role is to stimulate the hydrolysis of acetylcholine (ACh) to choline, and ACh deficiency is involved in the pathogenesis of AD. Inhibition of cholinesterase in the brain serves as a strategy to treat various cognitive diseases such as AD, senile dementia, ataxia, myasthenia gravis, glaucoma, and PD. Another important component in these pathologies is OS, which is responsible for extensive cellular free radical damage [43]. Several pharmacological studies focused on AChE inhibitors to reduce cholinergic deficits and improve neurotransmission in AD patients. However, these inhibitors were found to have certain limitations (e.g., short half-lives and associated hepatotoxicity), and current research aims to find new anti-AChE agents.

The inhibitory effects of the essential oil obtained from the bay laurel (LNEO) and rivastigmine (control) were tested against AChE [44]. The inhibition of AChE activity was determined spectrophotometrically (λ = 412 nm), by using an adaptation of the method previously described by Ingkaninan et al. [45], and results were expressed as IC50. Rivastigmine showed high anti-AChE activity and EOLn showed significant anti-AChE activity. This could be related to its main LNEO compound (eucalyptol), although anti-cholinesterase properties were reported for other terpenes too. The mechanisms of action involve competitive inhibition of the enzyme following the binding of bioactive molecules to the enzyme active site [44].

Yakoubi et al. [46] reported the chemical composition and synergistic interactions of Algerian essential oils of *L. nobilis* L. (LNEO), *Lavandula stoechas* L. (LSEO), and *Mentha pulegium* L. (MPEO) in terms of their anticholinesterase and antioxidant activities. The most abundant components of the analyzed EOs belonged to the class of oxygenated monoterpenes. LSEO showed interesting AChE- and butyrylcholinesterase (BChE)-inhibitory activity, while its binary combinations with LNEO had a synergistic effect on both enzymes. As for antioxidant activity, LNEO showed significant antioxidant activity [46].

An analysis of the potential neuroprotective activity of various leaf extracts was performed in a study by Duletić-Laušević et al. [47]. The highest AChE inhibition (%) was obtained by the water extract of commercial *L. nobilis* (90.72%), followed by acetonic and methanolic extracts. Since the acetone extract of *L. nobilis* showed the highest antioxidant activity (total phenolic content, TPC) and α-glucosidase inhibition, while the water extract of commercial bay laurel exhibited the highest AChE inhibition, the bay leaves could be a promising antioxidant, neuroprotective, and antidiabetic agent [47].

Caputo et al. [48] investigated the cytotoxicity of the bay laurel essential oil (LNEO) against the human neuroblastoma cell line (SH-SY5Y) cell line and the influence of LNEO on the expression of adenylate cyclase 1 (ADCY1), suggesting possible effects of essential oil on the CNS. The cytotoxic activity of 1,8-cineole and essential oil was investigated in the SH-SY5Y. IC50 values were >400 µg/mL, indicating that the substances were not cytotoxic, as per the National Cancer Institute criterion (substances with IC50 < 20 µg/mL were considered cytotoxic against treated cells). The authors also investigated the possible influence of essential oil and its major components on ADCY1 expression. The results showed that treatment with 200 µg/mL and 100 µg/mL of LNEO reduced ADCY1 expression in SH-SY5Y cells and, consequently, intracellular production of cAMP. 1,8-cineole had no effect on ADCY1 expression; thus, the effect of LNEO could be due to the presence of linalool, which has a dose-dependent sedative effect in the CNS. Moreover, regarding the complex phytochemical composition of essential oil, one or more components could act synergistically to affect ADCY1 expression in SH-SY5Y cells [48].

1,8-cineole (also known as eucalyptol) is a natural monoterpene compound and the main constituent of the essential oil of *L. nobilis* essential oil (LNEO). It exhibits numerous pharmacological effects, including anti-inflammatory and antioxidant activity, mainly via the regulation of NF-κB and Nrf2. It was verified to be ameliorated by 1,8-cineole on Ab25-35 in pheochromocytoma cells, and the anti-inflammatory mechanism may be related to NF-κB. Since OS is involved in the pathogenesis of AD, the antioxidant effect of 1,8-cineole might also contribute to the treatment. After pre-treatment with 1,8-cineole, the balance between oxidants and antioxidants was maintained by Nrf2-activated ROS scavenging effects. The effects of 1,8-cineole on the early stage of AD is mainly through inhibition of AChE, although the mechanism is unclear. However, studies showed that 1,8-cineole-rich plant extracts were more effective than pure 1,8-cineole in preventing AD. This may be due to the numerous components in the natural extract that produce synergistic effects. Based on previous research, 1,8-cineole is a promising drug for the prevention and treatment of AD due to its anti-inflammatory, antioxidant, and anti-AChE effects. However, investigations were limited to in vitro studies. Another limiting step in the treatment of AD is the ability to ensure the sufficient delivery of 1,8-cineole to the brain by crossing the blood–brain barrier [49].

Alkaloids represent an important active source in the search for compounds that act in the treatment of NDDs, as they were shown to have antioxidant and AChE-inhibitory properties. It was found that the predominant alkaloids in this group belong to the class of bisbenzylisoquinolines (BIS-BIQ) and that several factors play a crucial role in the AChE-inhibitory potential of BIS-BIQ alkaloids, including stereochemistry, the number of bridges, and the nature and position of substituents. It was found that the fewer the number of binding bridges between the molecules, the greater is efficacy, as demonstrated in the case of lindoldhamine, the first compound from the BIS-BIQ group to be evaluated for its AChE-inhibitory activity [50].

Acid-sensing ion channels (ASICs) are present in almost all types of neurons and play an important role in physiological and pathological processes. Recent literature data suggested that ASIC1a subtype is involved in the development of age-related diseases (e.g., stroke, rheumatoid arthritis, HD, and PD). The ASIC1a is the most sensitive channel to the medium’s acidification, and it is important for the excitation of neurons in the CNS. Ligands of the ASIC1a channel are a very interesting research area, both from fundamental and pharmaceutical aspects. Osmakov et al. [51] characterized lindoldhamine (LIN) from the leaves of *L. nobilis* as a novel inhibitor of the ASIC1a channel. LIN significantly inhibited the ASIC1a channel’s response to physiologically relevant stimuli in vitro, and in mice, intravenous administration of LIN at a dose of 1 mg/kg significantly reversed complete Freund’s adjuvant-induced thermal hyperalgesia and inflammation [51]. Treatment with LIN (1 mg/kg, i.p.) remarkably reversed CFA-induced thermal inflammation by reducing edema and hyperalgesia, but had no apparent analgesic effect on acetic acid-induced pain. More importantly, LIN could effectively inhibit ASIC1a activity in mild acidosis by 98.1%, 93.7%, and 69.3% at pH values of 6.9, 6.7, and 6.5, respectively. Thus, LIN showed a potent inhibitory effect on ASIC1a and a strong anti-inflammatory effect, but with no analgesic activity. Therefore, LIN can be considered as a novel antagonist of ASIC1a, which offers a new perspective for the treatment of inflammatory diseases as well as for the development of candidate drugs for the treatment of NDDs [52,53]. The anticholinergic activity was studied in the essential oil (LNEO), ethanolic extract, and decoction of bay laurel. Their activity against AChE was analyzed and showed an AChE-inhibitory capacity of more than 50% in the LNEO fraction and a high inhibitory value of AChE in the ethanolic fraction (64%). Thus, it can be concluded that *L. nobilis* has significant pharmacological potential in vitro and in vivo for the treatment of various diseases and was shown to be safe [42].

#### 3.1.2. *Laurus nobilis* and Parkinson’s Disease

Sesquiterpene lactone spirafolide, purified from the leaves of *L. nobilis*, inhibits ethanol increase in the blood of rats and the production of NO. It was shown also that has antimycobacterial properties. Spirafolide also inhibits the intracellular ROS and apoptosis in dopamine-induced neuroblastoma SH-SY5Y. This is important because ROS leads to abnormalities in the nervous system, as observed in AD and PD. Therefore, preventing the production of ROS by a naturally occurring small molecule may be a useful strategy to treat these NDDs. Pre-treatment with spirafolide significantly decreased both ROS production and apoptosis. The results indicate that different concentrations of spirafolide can reduce dopamine-mediated apoptosis. Considering these results, spirafolide from *L. nobilis* should be a potential candidate for the treatment of NDDs [54].

Reynosin, a sesquiterpene lactone isolated from the leaves of *L. nobilis*, was reported to have pharmacological effects, such as a reduction in ethanol concentration in rat blood and the production of NO. The aim of the study conducted by Ham et al. [55] was to determine whether reynosin protects neuronal cells from dopamine toxicity by regulating E6-AP and ASYN protein expression. The study was conducted in human dopaminergic cells and 6-hydroxydopamine (6-OHDA)-induced lesioned rat, as in vitro and in vivo models of PD, respectively. The results suggested that reynosin protects against dopamine-induced cell death and the resulting decrease in tyrosine hydroxylase (TH)-positive neuronal loss in 6-OHDA-lesioned rats through the down-regulation of ASYN concomitant with the up-regulation of E6-AP protein expression. The results presented suggest that reynosin may be a potential candidate for the treatment of NDDs such as PD [55].

Mansour et al. [42] provide an overview of the physiological properties and chemical composition of bay laurel. They reported the bay’s neuroprotective and anticholinergic activity. The effects of n-hexane fraction from *L. nobilis* leaves on dopamine-induced intracellular ROS production and apoptosis in human neuroblastoma SH-SY5Y were investigated. Compared with apomorphine (APO, IC50 = 18.1 μM) as a positive control, the IC50 value of the hexane fraction for dopamine-induced apoptosis was 3.0 μg/mL, and the values for the two major compounds, costunolide and ehydrocostus lactone, were 7.3 μM and 3.6 μM, respectively. The hexane fraction and these major compounds significantly inhibited ROS generation in dopamine-induced SH-SY5Y cells. To investigate the potential neuroprotective effects of the hexane fraction in vivo, a rodent 6-OHDA model PD was used. 6-OHDA was injected into the *substantia nigra* of young adult rats, and immunohistochemical analysis was performed to quantify TH-positive neurons. The hexane fraction significantly inhibited 6-OHDA-induced TH-positive cell loss in *substantia nigra* and also reduced dopamine-induced α-synuclein formation in SH-SY5Y cells, showing neuroprotective properties [42].

**Table 1:** *Laurus nobilis* bioactive components with neuroprotective potential.

*Laurus nobilis* Phytochemical Preparation or Compound	Action	Effect	Condition	References
Eucalyptol (1,8-cineol)	Regulation of NF-κB and Nrf2	Anti-inflammatory	AD	[49]
Quercetin	Modulation of Nrf2 expression Suppression of NF-κB signal transducer Destabilization and enhance of the clearance of abnormal proteins	Anti-inflammatory Antioxidant	NP	[56]
Spirafolide	Inhibition of intracellular ROS generation Inhibition of apoptosis in DA neuroblastoma SH-SY5Y cells	Neuroprotective Antioxidant	AD	[54]
Reynosin	Protection of DA induced cell death in neuroblastoma SH-SY5Y cells and 6-OHDA-lesioned rats Up regulation of E6-AP expression Down regulation of ASYN expression	Neuroprotective	PD	[55]
Lindoldhamine	Antagonist ASIC1a AChE inhibition	Anti-inflammatory	Ischemic brain damage AD	[40,51,52,53]
Ethanolic extract	Oxidative damage protection	Neuroprotective	Neurotoxicity of heavy metals	[57]
Polyphenol enriched leaf extract	Oxidative damage protection	Neuroprotective Antioxidant	AD	[58]
Water extract	AChE inhibition	Antioxidant Neuroprotective	AD	[47]
Apolar extract	Cytotoxicity and apoptosis on human neuroblastoma cells and rat glioma cells	Cytotoxicity	Neuronal cancer	[40]
Essential oil	AChE inhibition Reduction in ADCY1 expression in SH-SY5Y cells	Antioxidant Neuroprotective	AD	[46,48,49]
Essential oil	Modulation of glutamatergic and GABAergic transmission	Anticonvulsant	Epilepsy	[59]

Abbreviations: AD, Alzheimer’s disease; PD, Parkinson’s disease; 6-OHDA, 6-hydroxydopamine; AChE Acetylcholinesterase, ADCY1, adenylate cyclase 1; ASIC1a, acid-sensing ion channel 1a; α-SYN, α-synuclein; DA, dopamine; E6-AP, E6 associate protein; NF-κB, nuclear factor kappa light chain enhancer of activated B cells; Nrf2, nuclear factor erythroid 2–related factor 2; ROS, reactive oxygen species; SH-SY5Y, human neuroblastoma cell line (ATCC CRL-2266).

### 3.2. Laurus nobilis and Other Neurological Conditions

The possible protective effect of the essential oil of *L. nobilis* (LNEO) leaves against seizures induced by maximal electroshock (MES) or pentylenetetrazol (PTZ) was investigated to clarify the traditional belief in the antiepileptic effect of this plant. LNEO was isolated by a hydrodistillation procedure using Clevenger apparatus. GC-MS was used to characterize and identify the chemical compounds of LNEO. The study showed that LNEO has a protective effect against seizures induced by PTZ. In the anticonvulsant doses, LNEO showed sedation and motor impairment. Among the monoterpenes present in LNEO, eucalyptol, eugenol, methyleugenol, sabinene, terpineol, and pinene were the major ones. Moreover, researchers reported anticonvulsant activity of monoterpenes. Linalool is another monoterpene compound that has protective effects against PTZ-, picrotoxin-, and N-methyl-D-aspartate (NMDA)-induced convulsions. In addition, pinene, eugenol, and methyleugenol from LNEO showed an anticonvulsant profile in experimental seizures, such as the PTZ test. Modulation of glutamatergic and GABA-ergic transmission are some of the mechanisms in favor of the anticonvulsant action of monoterpenes such as linalool and eugenol. Thus, several mechanisms may be involved in the anticonvulsant activity of essential oils, which may be exerted by different components. The study found that LNEO produced sedation and motor deficits at the anticonvulsant doses. Eucalyptol was shown to have an inhibitory effect on locomotion and potentiate pentobarbital sleep time in mice. In addition, eugenol and methyleugenol exhibited anesthetic and muscle relaxant effects, and so, the observed sedation and motor impairment appear to be related in part to these components of LNEO. Further studies are needed to clarify the exact mechanisms and active compounds involved in these pharmacological effects [39,59]. To evaluate the neuroprotective properties of plant extracts from herbal drugs commonly used in traditional Mediterranean medicine, bay laurel leaves were studied. Cho et al. [60] found that the chloroform fraction of *L. nobilis* was able to protect against neuronal damage caused by cerebral ischemia. The inhibitory effect of spirafolide [54] and 3a-acetoxyeudesma-1,4(15),11(13)-trien-12,6a-olide [61] from bay laurel leaves on dopamine-induced apoptosis in dopaminergic SH-SY5Y cells was also observed.

Pacifico et al. [58] investigated the neuroprotective potential of *L. nobilis* antioxidant polyphenol-enriched leaf extracts. In this context, an alcoholic extract of bay laurel leaves and seven fractions obtained from it were tested. All extracts prepared by extractive and chromatographic techniques were phytochemically analyzed by the methods GC-MS and LC-MS. The potential antioxidant activity of the extracted fractions was investigated using DPPH^•^ and ABTS^•+^ assays, as well as specific assay media characterized by the presence of highly reactive ROS and RNS species (ROO^•^, OH^•^, O_2_^•−^, and NO). To evaluate the preparation of safe and nontoxic extracts, MTT, SRB, and LDH assays were performed with the human neuronal cell lines SH-5YSY and SK -N- BE (2)-C and with the C6 mouse glial cell line. Apoptosis-inducing properties were also assessed by spectroscopic evaluation of the ability of the extracts to activate caspase-3 and by DNA fragmentation assay. The data obtained in this way showed that the phenol-rich fractions (LnM, LnM-1, LnM-1a, LnM-1b, and LnM-2c) did not induce toxic effects while exerting significant cytoprotective and antioxidant responses in hydrogen peroxide- and Aβ(25-35)-fragment-oxidized cell systems. The potential antiamyloidogenic activity of *L. nobilis* leaf polar extracts in the Aβ(25-35) fragment-oxidized cell systems was further analyzed by Congo red staining. It could be concluded that the use of plant ingredients could be a valuable strategy to counteract the progression of neurodegeneration phenomena [58].

The polyphenolic profile of *L. nobilis* leaves includes compounds belonging to the classes of phenolic acids, flavonols, flavan-3-ols, flavones, and proanthocyanidins. Flavonols are the most abundant phenolic group and consist mainly of kaempferol and quercetin glycosides [62]. Quercetin is a widely distributed flavonoid, a major component of the flavonol subclass. It is commonly found in vegetables, fruits, nuts, and grains in association with sugars, phenolic acids, and alcohol, and accounts for 60–75% of the total flavonoid intake. Numerous pharmacological applications of quercetin, including antioxidant, neuroprotective, antiviral, anticancer, cardiovascular, antimicrobial, anti-inflammatory, hepatoprotective, and anti-obesity effects, made it a promising phytochemical for the prevention of lifestyle-related diseases. The protective effects of quercetin in the treatment of NDDs and cerebrovascular diseases were demonstrated both in vitro and in vivo studies. Quercetin acts as an effective therapeutic agent against various NDDs by suppressing OS, inflammation and promoting neurogenesis. It was also reported to have the ability to reverse cognitive impairment and improve memory performance during aging. As a potent antioxidant, quercetin increases the survivability of neurons against a variety of oxidative damage. The presence of a certain number of free hydroxyl groups in the chemical skeleton of quercetin plays an important role in its antioxidant activity. The direct and indirect antioxidant activity and the ability of quercetin to chelate metals play a key role in attenuating ROS-mediated neuronal injury. Quercetin attenuates inflammation-induced neuronal damage by downregulating the expression of proinflammatory cytokines and chemokines, such as COX. Quercetin also promotes neurogenesis by stimulating the sirtuin (SIRT1) and cAMP- response element binding protein (CREB) pathways, thereby increasing the level of neurotrophic factors important for neurogenesis. The ability of quercetin to prevent the aggregation and disaggregation of abnormal proteins such as Ab-peptide and a-synuclein makes it an effective drug for the treatment of NDDs such as AD and PD. Quercetin also plays an important role in neuroprotection and can be considered as an effective nutraceutical agent with limited toxicity. Although quercetin shows multipotent neuroprotective effects in various in vitro and in vivo neurodegenerative model systems, its application in the pharmaceutical field is limited due to its poor solubility, bioavailability, and instability. In addition, the scientific evidence on the clinical trials and toxicity of quercetin is insufficient. Therefore, future research needs to focus on (1) improved drug delivery systems (e.g., prodrugs, nanoencapasulation, and microemulsion) to increase bioavailability and blood–brain permeability; (2) further clinical trials to determine the effective dose for the treatment of NDDs; (3) additional analysis of the distribution of quercetin metabolites in the CNS of experimental models of NDDs; and (4) current in vivo toxicity profiles to evaluate neurotoxic effects [56,63].

Two clinical studies concerning the antioxidant and anti-inflammatory effects of *L. nobilis* were registered and are ongoing, but the results are not yet available, i.e., at the time of this review. Considering the previous results of scientific studies presented in this review, the authors conclude that laurel-based phytopreparations can help in the treatment of neurological disorders and also suggest further phytochemical and clinical research of bay laurel with the aim of confirming its phytotherapeutic potential for the treatment of NDDs.

## 4. *Aronia melanocarpa* (Rosaceae)

The Rosaceae plant family is one of the largest and most important families of flowering plants, with over 3000 species worldwide. It includes popular fruit trees such as apples, peaches, plums, cherries, and almonds, as well as ornamental plants such as roses, cotoneasters, and photinias. *Aronia melanocarpa* (Michx.) Elliott 1821, also known as black chokeberry, is a deciduous shrub that belongs to the Rosaceae family. It is native to North America and is commonly found in moist forests and along streams [64,65]. The shrub is known for its small, white flowers that bloom in spring and its edible fruits that are rich in antioxidants and vitamins. The fruit has a tart flavor, and although the berry can be eaten unprocessed, it is more commonly used to make juices, jams, jellies, and syrups. Aronia berry is well known for its numerous health benefits, which are mainly attributed to its high content of polyphenols. In addition to polyphenols, the functional components of aronia berry consist of nutrients, fiber and sorbitol, while organic acids, proteins, and lipids are responsible for the quality and stability of this fruit [65].

Compared to most vegetables and fruits, including fruits from the Rosaceae family, aronia berries contain significantly more polyphenols, including anthocyanins, proanthocyanidins, flavonols, and phenolic acid [66,67]. These compounds contribute to the health benefits of aronia berries but are also responsible for the taste, which limits consumer appeal due to pronounced astringency and bitterness.

The most abundant polyphenols in aronia berry are anthocyanins, a group of water-soluble pigments responsible for the dark purple, blue, and red colors of many fruits and vegetables, including aronia berry. In aronia berry, anthocyanins act as a natural sunscreen, protecting the plant from damage caused by the sun’s UV rays [68]. They also play a role in attracting pollinators, as the bright colors are a visual signal to insects, and most importantly, they are powerful antioxidants that protect the plant from OS and free radical damage. These antioxidant properties are also beneficial to humans, as consumption of anthocyanin-containing foods was shown to reduce the risk of chronic diseases such as cardiovascular disease, type 2 diabetes, and cancer. Aronia berry also contains proanthocyanidins, which are believed to have numerous health benefits for humans. Like anthocyanins, proanthocyanidins have antioxidant properties and can protect the body from OS and free radical damage. The anti-inflammatory effects of proanthocyanidins may be responsible for improving cardiovascular health by increasing blood vessel flexibility and reducing oxidative damage to LDL cholesterol [65]. Less abundant compounds in aronia berry are flavonols, including quercetin, and phenolic acids, while it also contains many so-called non-extractable polyphenols.

### 4.1. Aronia berries in Neurological Disorders: What Are the Potential Mechanisms for the Neuroprotective Actions?

As mentioned earlier, previous studies showed some interesting preventive and therapeutic effects of aronia berries that could be used for the treatment of noncommunicable diseases that are the most common causes of morbidities today—cancer, diabetes, and cardiovascular diseases [67,69,70,71,72]. In addition, aronia berries were studied and were shown to have potential neuroprotective properties. Although studies on the effects of aronia berries in CNS disorders are not as numerous compared to the previously mentioned disorders, the results of the research efforts conducted to date seem promising. These studies include testing the effects of consumption of dietary products obtained from the berries [73,74,75], or the use of aronia extracts [76], isolated polyphenols abundant in this fruit [77], as well as the studies using specific compounds, e.g., specific polyphenols or their metabolites [78,79,80]. Aronia berries’ effects were studied using in vitro and in vivo models of human neurological diseases, as well as in some human clinical trials [81].

**Figure 4:**
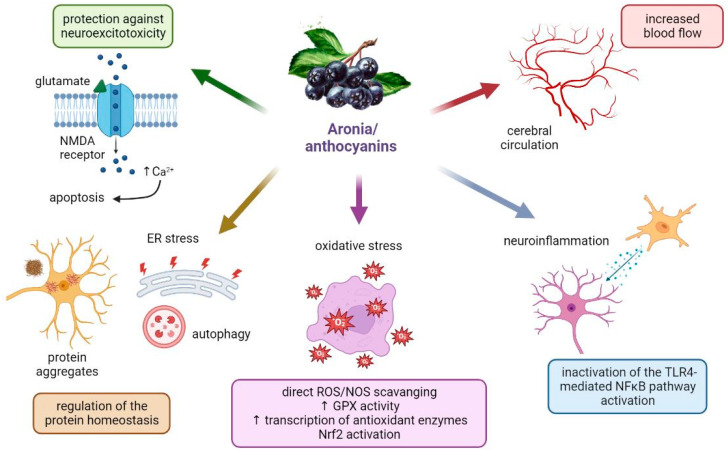
Proposed mechanisms by which *Aronia melanocarpa* berry and its major components, anthocyanins, exert neuroprotective effects. It is thought to involve multiple processes, including antioxidant activity, anti-inflammatory effects, reduction in protein aggregation, increased blood flow to the brain, and protection against neuroexcitotoxicity. Abbreviations: NMDA, N-methyl-D-aspartate receptor; ER, endoplasmatic reticulum; GPx, glutathione peroxidase; ROS, reactive oxygen species; NOS, reactive nitrogen species; Nrf2, nuclear factor erythroid 2–related factor 2; TLR4, Toll-like receptor 4; NFκB, nuclear factor-κB.

With regard to elucidating the exact way in which aronia extracts and their major components (the most studied being anthocyanins) act neuroprotectively, multiple mechanisms likely act together to produce ameliorative effects in the CNS, including slowing the progression of NDDs (Figure 4).

It is believed that the mechanism of antioxidant action of aronia berries, as the best known and proven effect, involves several different processes. It is known that the components of aronia berry act as radical scavengers, reduce OS by regulating the production of ROS/RNS, and maintain the balance of antioxidants in the body.

Some of the proposed mechanisms of antioxidant action of aronia are shown in Figure 4. The major antioxidants in aronia berry are vitamin C and various polyphenols, such as anthocyanins, phenolic acids, flavanols, flavonols, and tannins. From the in vitro studies, including tests such as DPPH assay, TEAC method, and ABTS radical assay, it was found that incubation with bioactive chokeberry compounds causes an increase in antioxidant defense, and it is believed that a decrease in ROS/RNS levels is the result of direct scavenging properties. In vivo studies on bioactive compounds from aronia berries are limited. Aronia-derived anthocyanins were found to reduce lipid peroxidation [82]. However, the antioxidant effect of aronia extracts in vivo is not limited to free radical scavenging, but also includes the suppression of ROS/RNS formation, restoration of antioxidant enzyme expression and activity, as well as changes in cellular signaling in terms of enhancement of antioxidant defenses [83]. For example, the activities of all antioxidant enzymes, namely SOD, CAT, and GPx, were found to increase upon supplementation with aronia extract [76]), as it was found to upregulate antioxidant gene expression. The same study discovered increased gene expression of proteins belonging to the heat shock family, a group of molecular chaperones that combat oxidative and thermal stress and are involved in lifespan and stress resistance during aging.

Other proposed mechanisms of the protective effects of aronia products include reducing neuroinflammation, regulating protein synthesis, combating neuroexcitotoxicity, and increasing cerebral blood flow. Some of the phytochemicals in aronia berries were shown to have anti-inflammatory effects, which may additionally help reduce OS and protect against cellular damage. Black chokeberry extract was found to significantly reduce the generation of NO, decrease the production of numerous inflammatory factors such as TNF-α and IL-2, attenuate the activation of the NF-κB pathway, and suppress the expression of iNOS and COX-2 [84,85,86,87,88]. In addition, bioactive substances in chokeberry may provide protection by not only reducing OS and inflammation but also improving blood flow [89,90]. In neurons, excitotoxic stimuli cause massive calcium influx, stimulate ROS/RNS production, and contribute to the activation of pro-death factors such as calpains. Through a yet unknown mechanism, anthocyanins appear to be able to maintain calcium homeostasis. Studies showed that they are able to reduce calcium influx [84,91]. Recent research also suggested that anthocyanins may be able to directly prevent protein aggregation and stimulate autophagy, as, for example, pure anthocyanin cyanidin-3-O-glucopyranoside was found to directly interfere with Aβ-peptides [92].

An important prerequisite for the use of dietary supplements for the purpose of their health benefits is that the active ingredients are able to reach the target tissue in the desired concentrations. It is well known that the overall bioavailability of anthocyanins, including those contained in chokeberry extract, is quite low. Studies showed that these molecules are already largely metabolized in the intestine, and only a small fraction of the molecules reach the target tissue in unchanged form. Nevertheless, the results of studies, in which anthocyanins or anthocyanin-rich products were used and showed significant biological effects, suggest that their metabolites probably have therapeutic properties. To date, most studies on the health-promoting potential of anthocyanins used unmodified molecules, including numerous in vitro studies. Therefore, it is crucial to include the major metabolites of this type of flavonols when studying their biological activities at the cellular level. Currently, there is evidence of the distinct antioxidant abilities of phenolic acid metabolites related to their neuroprotective effects [93,94,95]. In addition, protocatechuic acid (PCA), a major metabolite of cyanidin-3-glucoside, was found to inhibit the aggregation and fibril destabilization of amyloid-β and α-synuclein proteins, suggesting beneficial effects in reducing neuropathological changes in AD and PD, respectively [80]. It was also found to reduce α-synuclein toxicity in PC12 cells [96] and to have neuroprotective effects in a PD model in mice [97]. In addition to PCA [98], another anthocyanin metabolite, gallic acid, was shown to attenuate aluminum-induced neurotoxicity in rats, which is thought to be associated with the pathophysiology of several NDDs, including AD and ALS [99]. These are only selected studies that examined some of the products of the compounds found in aronia berries, as there are many others that evaluated the effects of the aforementioned molecules in various disease models, including those involving the CNS. For more information, reading the review by Winter and Bickford [24] is recommended, as it provided a much more detailed overview of this topic. Regarding the bioavailability of active substances from aronia berry, it should be added that it can be affected by various pharmaceutical interventions, such as the microencapsulation or the nanoformulation of anthocyanins [100]. These methods could improve the stability of the compounds, and encapsulation could improve their intestinal absorption and metabolism.

Regarding the research on the effects of aronia extract, purified anthocyanins, or their metabolites in in vitro studies, it is important to emphasize that the use of cell culture platforms for evaluating the potential of these compounds in attenuating cellular damage offers certain advantages. However, the disadvantage is that the concentrations used for in vitro studies certainly cannot be achieved in target tissues under physiological conditions. Nevertheless, it is important to point out that the use of cell lines in these studies is advantageous because they provide valuable information about the mechanisms of action of the compounds under investigation. In addition, compared to other similar dietary supplements, it was found that the anthocyanins from aronia, including their potentially biologically active metabolites, were found to appear highly unchanged in serum and urine, making it physiologically reasonable to use these products in isolated cells or tissues [101].

### 4.2. Aronia melanocarpa and Its Extracts in Neurodegenerative Diseases

Regarding potential neuroprotective effects in neurological diseases characterized by cognitive decline (i.e., AD and other dementias), some, albeit limited, studies suggested that aronia berry may help improve cognitive function and memory. In the scopolamine-induced memory impairment model in mice [102], aronia extract was administered orally to mice 120 min before water maze tests for 4 days and once for training in the passive avoidance test. The administration of chokeberry extract attenuated scopolamine-induced learning and memory impairment in the Morris water maze and passive avoidance test, decreased hippocampal AChE levels in scopolamine-injected mice, and increased hippocampal BDNF and p-CREB expression. In aged rats, i.e., 24 months old, aronia fruit juice had a neuroprotective effect and improved cognitive and locomotor functions [73]. In the hippocampus, this treatment did not affect the number of neurons in the dentate gyrus, but it significantly increased the density of nerve fibers in the perforant path of this brain region. In addition, aronia juice added to the diet for 105 days increased AChE activity in the hippocampus, indicating improved functional activity of cholinergic neurons. Furthermore, other preclinical studies showed beneficial effects of isolated and purified anthocyanins in other models related to cognitive impairment (see Table 2).

Recently, several intervention studies with aronia supplementation were conducted in humans, but few of them addressed the CNS effects of this underutilized but very promising functional food. In a randomized, controlled trial of healthy, overweight, middle-aged individuals, participants consumed *A. melanocarpa* extract for 24 weeks and then, underwent a series of tests of cognitive performance, mood, and vascular function [81]. The intake of aronia extract supplementation showed a positive effect on cognitive performance and blood pressure in these at-risk individuals.

The potential benefits of regular dietary intake of flavonoids, including chokeberry products, which are particularly rich in these compounds, were already suggested to reduce the risk of PD [109,110], and even reduce the risk of PD-related mortality [111]. Research also showed that various fruit and plant extracts rich in anthocyanins and proanthocyanidins have significant neuroprotective activity [112]. However, to the best of our knowledge, there is no research that specifically used aronia berry and its extracts in PD studies, either in vivo or in vitro, regardless of the undeniable potential of this berry and its associated products. In Table 2, we listed the results of a few studies in which some components of the aronia berry were used, particularly anthocyanin metabolites and PCA, which showed strong potential for use in PD therapy. There is evidence of the protective role of anthocyanins extracted from aronia berry against mitochondrial dysfunction [105]. Considering the fact that mitochondrial dysfunction plays an important role in the pathogenesis of PD, it is certainly interesting to investigate, in the future, whether there is a potential for the use of aronia berry and aronia-derived therapeutics for the prevention and treatment of this disease.

### 4.3. Aronia melanocarpa and Other Neurological Conditions

In addition to the NDDs mentioned above, the effects of aronia berries and their components were explored in some other CNS pathologies. For example, in adult rats, aronia reduced anxiety-like and depression-like behaviors after a month of unlimited drinking the diluted juice [75]. Aronia fruit juice exhibited anxiolytic effects evidenced by a dose-dependent increase in the time of active social contact between test partners, which was comparable to the effect of diazepam [74]. In a model of focal ischemia in mice, orally ingested cyanidin 3-O-β-galactoside reduced superoxide levels, infarct size, and improved neurological outcomes [79]. Given the growing evidence from preclinical and especially in vitro studies of the potential benefits of aronia berry, its extracts, and associated bioactive molecules, further research is needed to determine whether these results translate to clinical settings.

## 5. Celastrol

**Figure 5:**
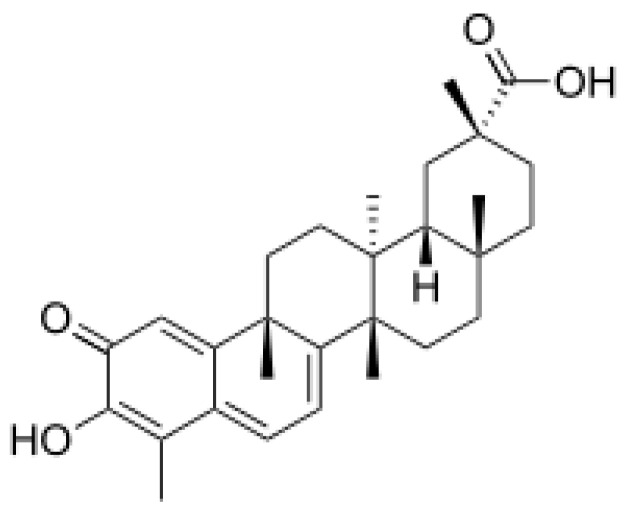
Chemical structure of celastrol.

Celastrol is one of the important bioactive components extracted from several autochthonous Chinese medicinal plants, including *T. wilfordii* (Lei Gong Tank; Thunder of God Wine) and *T. regelii* (Regel’s Three-Winged Nut, Yellow Wine). It belongs to the group of pentacyclic nortriterpenquinones (molecular formula C_29_H_38_O_4_) [113] (Figure 5). Celastrol has been used in traditional Chinese medicine for centuries to treat a variety of diseases, including inflammation, fever, asthma, and autoimmune diseases. In recent years, it was studied for its potential therapeutic effects in the treatment of several other diseases, such as neurological disorders, obesity, diabetes, and cancer. Due to its anti-inflammatory and antioxidant properties, as well as its ability to modulate the immune system, numerous experimental in vivo and in vitro studies were conducted to investigate its therapeutic potential in various NDDs [114].

### 5.1. Therapeutic Potential of Celastrol in Neurodegenerative Diseases

The potential neuroprotective effects of celastrol were investigated in various experimental in vivo and in vitro models of chronic NDDs, such as AD, PD, or ALS, as well as in acute neurological conditions that may lead to secondary neurodegeneration as described in focal or global cerebral ischemia (e.g., stroke). The mechanism of action of celastrol as a neuroprotective agent in the above NDDs was not fully elucidated. However, preclinical studies suggested that it exerts its neuroprotective effects through multiple mechanisms, mainly through its anti-inflammatory and antioxidant actions [115]. This is supported by the fact that it can suppress many steps in the initial stages of inflammation and OS, including signaling pathways for HSP90 and NF-κB, a transcription factor thought to play an important role in gene regulation during OS and inflammation. Recent studies showed that celastrol is not only a potent inhibitor of transcription factors, but also an inhibitor of inflammatory cytokines, including IL-1β and TNF-α in LPS-stimulated RAW264.7 cells [116]. Celastrol, as a potential drug, may inhibit the induction in iNOS and other genes, as well as the production of oxidants that cause neuron dysfunction or death. According to Alison et al., celastrol inhibits the peroxidation of the inner and outer mitochondrial membrane by binding oxygen free radicals and preventing them from attacking the inner membrane by increasing the negative surface charge [113]. Recently, much attention was paid to the modulation of the heat shock response as a potential model for the treatment of human diseases such as cancer, trauma, ischemia-reperfusion, transplant surgery, or diabetes [117]. Research data supported the cytoprotective effect of celastrol due to its mechanism of action, which involves the expression of HSPs such as HSP27, HSP40, and HSP70 [118]. The action of celastrol is thought to cause nuclear translocation of the transcription factor heat shock factor 1 (HSF1), which, in turn, leads to a heat shock response that induces the expression of HSPs. Furthermore, the neuroprotection of celastrol could be explained by the inhibition of apoptosis due to a decrease in caspase activation, inhibition of Aβ production, and modulation of autophagy [119].

The results of numerous studies on experimental models of AD showed that the potential mechanisms of celastrol neuroprotection are exerted through different pathways. The anti-inflammatory and antioxidant effects were modulated by increasing the expression of Bcl-2, such as HSP27 and HSP70, and decreasing the production of ROS [120]. Celastrol was found to reduce the expression of NF-κB, COX-2, and glycogen synthase kinase-3 beta (GSK-3β) as key regulators of the inflammatory response in microglia and neurons [121]. Low doses of celastrol were also reported to decrease the production of TNF-α and IL-1β by human monocytes and macrophages, the expression of major histocompatibility complex molecules II (MHC II) in microglia, and the production of iNOS in endothelial cells [122]. The main neuropathological markers of AD are the extracellular neuritic plaques and insoluble Aβ deposits, such as the accumulation of phosphorylated MAPT/tau (microtubule-associated protein tau) aggregates responsible for neurofibrillary tangles. In vitro and in vivo studies showed that celastrol inhibits Aβ production, partly due to reducing expression of the rate-limiting enzyme BACE-1 by preventing NF-κB activation [123]. Furthermore, celastrol promotes autophagy and lysosomal biogenesis in vitro and in the brain of P301S-MAPT/tau and 3XTg mice by activating TFEB (transcription factor EB), which effectively degrades MAPT/tau aggregates [124].

The results of numerous experimental studies also showed that celastrol administration prevents the loss of dopaminergic neurons in the substantia nigra and the reduction in striatal dopamine levels. The possible mechanisms of its neuroprotective effect in the models of PD are mainly related to the increased expression of HSP70 in neurons and the potent anti-inflammatory and antioxidant effects. the induction in HSP70 expression was shown to reduce the inflammatory response by preventing TNF-α and NF-κB activation [125]. Moreover, celastrol was shown to effectively reduce OS in a rotenone-induced PD model, exert anti-apoptotic effects in SH-SY5Y cells, and induce autophagy, which is critical for proteolytic degradation of α-synuclein and cleavage of damaged mitochondria [126]. According to Choi et al. [127], celastrol maintained mitochondrial function and inhibited p38 MAPK. Moreover, in a genetically modified MPTP-induced PD mouse model, celastrol improved motor deficits and prevented nigrostriatal dopaminergic degeneration via the Nrf2-NLRP3-caspase-1 pathway [97].

The potential neuroprotective effect of celastrol in the treatment of ALS is complex and mainly related to its anti-inflammatory, antioxidant, and anti-apoptotic properties [119]. In the SOD1 transgenic mouse model, celastrol improved the symptoms of ALS, by arresting spinal cord neuron loss, increasing HSP70 expression and decreasing TNF-α and iNOS levels in the spinal cord [125]. Similar results were described in several in vitro models of ALS, where celastrol exerted neuroprotective effects on primary motor neurons induced by staurosporine or H_2_O_2_ by activating HSP70 expression. Li HY et al. [128] demonstrated that celastrol reduced cell death by activating mitogen-activated protein kinase (MEK)/extracellular regulated protein kinases (ERK) and phosphoinositide 3-kinase (PI3K)/serine-threonine kinase (AKT) signaling pathways, in G93A- SOD1 transfected NSC34 cells, as a cellular model of ALS.

**Figure 6:**
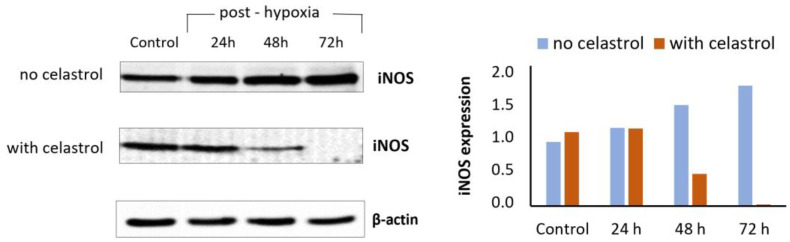
Expression of iNOS under hypoxic conditions and celastrol treatment. Western blot (immunoblot) assay with densitometric analysis revealed decrease in iNOS expression at 48 to 72 h post-hypoxia in BV-2 microglia cells that were pre-treated with 100nM celastrol following 6 h of hypoxia.

Celastrol was reported to have neuroprotective potential in different models of neuronal hypoxia/ischemia exerted either in vivo (transient middle cerebral artery occlusion model) or in vitro (oxygen-glucose deprivation/reoxygenation model) by various complex mechanisms. Different authors showed that celastrol reduces infarct volume and neuroinflammation, likely through the suppression of the high mobility group box 1 (HMGB1)/NF-κB signaling pathway [119,125,129]. Various experimental in vivo and in vitro models of hypoxia/ischemia showed that the antioxidant and anti-inflammatory effects of celastrol were mainly mediated by increasing the expression and activity of IL-33 and IL-10, SOD and GPx, Nrf2 and SIRT1 and SIRT3, and by decreasing inflammatory cytokines, such as IL-1β, I-6, and TNF-α, or prooxidants such as iNOS (authors’ preliminary data; Figure 6) and COX-2 [130,131]. Recently, the neuroprotective potential of celastrol was shown to be mediated by IL-33/ST2 axis-mediated M2 microglia/macrophage polarization and by the suppression of glycolysis through the inhibition of HIF-1α/PDK1, such as the inhibition of AK005401/MAP3K12 and the activation of the PI3K/AKT pathway. In recent experiments, Hong et al. showed that the decrease in OS and the resulting neuroprotective effect could be mediated by the binding of celastrol to the neuronally expressed developmentally downregulated 4 (Nedd4) to regulate the ubiquitylation of Nrf2 in astrocytes [132]. Although clinical trials of celastrol were conducted for other health conditions, there are no clinical studies to date to validate its potential neuroprotective effects. To ensure the appropriate translation of promising preclinical results to the clinical setting, the possible molecular targets, pharmacokinetic properties, and toxicological profile of celastrol should be further clarified.

### 5.2. Celastrol and Other Health Conditions

Due to complex mechanisms of action, the potential beneficial effects of celastrol were described in various health conditions, as well as in experimental models of various pathological conditions. In Chinese medicine, it is traditionally used to treat several chronic inflammatory diseases [114]. According to Schiavone et al., there are several clinical conditions such as Crohn’s disease, psoriasis, rheumatoid arthritis, diabetes, kidney disease, and transplantation with promising results of celastrol administration [115]. In vitro and in vivo animal studies suggested that celastrol has antidiabetic effects and improves diabetic nephropathy and whole-body insulin resistance, due to the inhibition of NF-κB in the hypothalamus [133]. In mice, celastrol showed obesity-controlling effects by inhibiting negative regulators of leptin [134], and reduced obesity-induced OS by increasing antioxidant enzymes and inhibiting NADH oxidase and ROS [135]. Moreover, celastrol was shown to effectively induce ROS accumulation and apoptosis by down-regulating HSP90 in tumor cells. Similarly, celastrol caused G2/M phase arrest in osteosarcomas and induced apoptosis and autophagy via the ROS/JNK signaling pathway [128]. Celastrol also enhanced the antioxidant defense system and provided protection against bleomycin-induced pulmonary fibrosis in rats by inhibiting antioxidant enzymes such as heme oxygenase-1 (HO-1), glutathione S-transferase, and NADPH: quinine oxidoreductase restored via the Nrf2 pathway [114]. Recently, it was revealed that it may have cardioprotective effects either directly or indirectly through complex and interlinked antioxidant and anti-inflammatory effects [136]. As an inhibitor of the ROS-generating protein NOX2, celastrol has beneficial effects on several cardiovascular pathologies, including obesity, diabetes, inflammation, atherosclerosis, valvular calcification, vascular and cardiac remodeling, and heart failure. Although these studies showed promising results, further research is needed to determine the safety and efficacy of celastrol, and the mechanism of action in the treatment of the above diseases.

## 6. Conclusions

This review summarized the main aspects related to the potential of using extracts of *L. nobilis* and *A. melanocarpa*, their related components, as well as celastrol, another plant-derived molecule, for the prevention and treatment of NDDs. The common characteristic of these selected plants and their associated molecules was found to be their complex mechanisms of action with predominantly antioxidant activity, which makes them promising in terms of their therapeutic potential. Despite the promising results of numerous in vitro and in vivo studies, their therapeutic potential still seems to be underestimated in science and medicine, especially when compared to their better-known counterparts such as green tea polyphenols, curcumin from turmeric, or resveratrol from grapes. We hope that this review is convincing enough to demonstrate the need for further research on the potential benefits of the reviewed plant-derived pharmaceuticals with pronounced antioxidant activity for the treatment of NDDs.

## Figures and Tables

**Table 2:** Selected recent preclinical studies on the neuroprotective effects of aronia berry extracts, anthocyanins, and their metabolites in experimental models associated with Alzheimer’s and Parkinson’s diseases.

Experimental Model	Treatment	Outcomes	Reference
In vivo studies			
amyloid-β-induced memory damage in rats	isolated and purified anthocyanins	higher free radical scavenging abilities than the crude anthocyanins extract, improved spatial memory in a Morris water maze test, protection of the cells in the hippocampus against Aβ toxicity	[103]
D-galactose model of accelerated aging in mice	anthocyanins isolated as pure compounds (cyanidin 3-arabinoside, cyanidin 3-galactoside, cyanidin 3-xyloside, and cyanidin 3-glucoside), low (15 mg/kg) and high (30 mg/kg) dose	anthocyanins blocked age-associate cognitive decline and response capacity, improved redox system balance, decreased inflammatory cytokines’ levels, decreased DNA damage	[77]
amyloid-β-intracerebral injection induced cognitive impairment in rats	cyanidin 3-O-glucoside	protective against the Aβ-induced impairment, but no effect on normal learning and memory; attenuated Aβ-induced tau hyperphosphorylation and GSK-3β hyperactivation	[78]
experimental dementia model in rats by p.o.aluminium chloride application	protocatechuic acid	treatment significantly modulated memory deficits, via anti-cholinesterase, anti-oxidative and anti-inflammatory activities	[98]
senescence accelerated (SAMP8) aging mice	intragastric cyanidin 3-O-β-galactoside from black chokeberry (25 and 50 mg/kg/day) for 8 weeks	alleviated decline in brain glucose uptake, neuronal damage in the hippocampus and cortex, reduced β-amyloid load in the brain and significantly increased the crossing-platform number in the Morris water maze test	[104]
In vitro studies			
PC12 cells exposed to amyloid-β and α-synuclein	protocatechuic acid	inhibition of fibril formation, destabilization of preformed fibrils of amyloid-β and α-synuclein	[80]
amyloid-β-induced apoptosis of SH-SY5Y cells	*A. melanocarpa* anthocyanins pretreatment	inhibited apoptosis, regulated Ca^2+^ homeostasis and apoptosis-related genes and inhibited mitochondrial dysfunction	[105]
α-chymotrypsin amyloid like fibrils formation model	chokeberry juice	inhibited aggregation of α-chymotrypsin	[106]
primary neuronal cells exposed to hydrogen peroxide or amyloid-β	ethanolic extract from black chokeberry fruit	reduced amyloid-β-induced neuronal cell death by modulating the inflammation-related signaling pathways	[107]
MPTP model of PD in mice	protocatechuic acid	improved rotarod test, ameliorated SN pathology and DA levels	[97]
PC12 cells exposed to α-synuclein	protocatechuic acid, hydroxytyrosol	decreased toxicity of α-synuclein, increased the expression vitagenes system proteins	[96]
neuroblastoma cells exposed to paraquat	aronia berry concentrate	protection of neurons from cell death, attenuated increase in superoxide, hydrogen peroxide, and oxidized glutathione levels	[108]

Abbreviations: GSK-3β, Glycogen synthase kinase-3 beta; DA, dopamine; MPTP, 1-methyl-4-phenyl-1,2,3,6-tetrahydropyridine; PD, Parkinson’s disease; SN, *substantia nigra.*

## Data Availability

Data sharing not applicable.
